# The effects of interventions preventing self-harm and suicide in children and adolescents: an overview of systematic reviews

**DOI:** 10.12688/f1000research.19506.2

**Published:** 2020-02-18

**Authors:** Ida Sund Morken, Astrid Dahlgren, Ingeborg Lunde, Siri Toven

**Affiliations:** 1 Regional Centre for Child and Adolescent Mental Health, Eastern and Southern Norway (RBUP), Oslo, Oslo, 0484, Norway; 2 Regional Centre on violence, trauma and suicide prevention, Eastern Norway, Oslo, Oslo, 0484, Norway

**Keywords:** Self-harm, Suicide*, Adolescents, Children, Mental health, Prevention, Treatment, Evidence-based practice

## Abstract

**Background: **Self-harm and suicide in children and adolescents are of serious consequence and increase during the adolescent years. Consequently, there is need for interventions that prevent such behaviour. The objective of this paper: to evaluate the effects of interventions preventing self-harm and suicide in children and adolescents in an overview of systematic reviews.

**Methods:** We conducted an overview of systematic reviews (OoO). We included reviews evaluating any preventive or therapeutic intervention. The methodological quality of the included reviews was assessed independently, and data was extracted by two reviewers. We report the review findings descriptively. The certainty of evidence was assessed using Grading of Recommendations Assessment, Development and Evaluation (GRADE).

**Results: **Moderate certainty evidence suggests that school-based interventions prevent suicidal ideation and attempts short term, and possibly suicide attempts long term. The effects of community-based interventions following suicide clusters and local suicide plans are unknown, as are the benefits and harms of screening young people for suicide risk.

The effects of most interventions targeting children and adolescents with known self-harm are unknown. However, low certainty evidence suggests that dialectical behavioural therapy and developmental group therapy are equally as effective on repetition of self-harm as enhanced treatment as usual.

**Conclusions: **Research on several recommended practices, such as local suicide plans, prevention of suicide clusters and approaches to risk assessment, is lacking. When such interventions are implemented, the effects should be closely evaluated. There is also need for more research on treatment of repeated self-harm. Further research should include long term follow-up, and investigate possible adverse effects.

In prevention of self-harm and suicide in children and adolescents, policy makers and health providers should consider evidence from population-based studies with mixed-age samples, adult samples, and studies on conditions associated with self-harm and/or suicidality, such as depression and psychosis.

**PROSPERO registration**: CRD42019117942 08/02/19

## Introduction

Self-harm involves intentional self-poisoning or self-injury, irrespective of type of motive or the extent of suicidal intent ^1, 2^. It is often a coping mechanism used to solve a difficult situation, and can serve functions such as affect regulation, communicating the extent of pain, or self-punishment ^3^. While self-harm is rare in children younger than 12 years ^4^, it is prevalent amongst adolescents ^5^: across international studies, 18% between the ages of 12 and 18 report a history of one or several episodes of intentional self-harm. Prevalence is highest amongst adolescent girls, typically done by cutting, but self-harm is also a problem amongst boys, more often hitting themselves ^6, 7^. It may be temporary or more long-lasting in nature ^6^, and one episode of self-harm is a strong predictor of repetition ^8, 9^. When repeated, the person often advances to a combination of different methods, increasing the medical severity ^10^. Completed suicide is on the other hand defined as the act of intentionally ending one **’**s own life ^11^. Suicide is rare before the age of 15 but increases in prevalence through adolescence ^5^, and is somewhat most prevalent amongst males ^12^. It is the most common cause of death in female adolescents, and the third most common cause of death in male adolescents (after road-traffic accidence and violence) ^5^. Self-harm and suicide result from underlying risk- and maitaining factors, spanning from other mental health problems such as depression, biological factors, exposure to traumatic events or other difficult circumstances in the young person’s environment ^4, 13^. Furthermore, there are repercussions to being exposed to family and/or friends’ self-harm and suicide. Such exposure may contribute to self-harm and suicide in adolescents, a phenomenon referred to as “social contagion” ^4^. Related, the bereavement process of survivors after losing a significant other may last a long time and increase the risk of suicide ^14^ and suicidal thoughts ^15^.

Evidently, self-harm and suicide in children and adolescents are complex and multifaceted phenomena. As prevention likely warrants a variation of measures, clinicians and policy makers are in need of knowledge the effects of different types of preventive interventions.

There are several reviews with summarized evidence on effects of interventions aimed at preventing (re)occurance of self-harm and suicide. However, many reviews are of variable quality, or outdated ^16– 21^. Furthermore, there is a large overlap of interventions covered in the different reviews, making it difficult for professionals to sort out the best available evidence needed to make informed decisions ^22^. Consequently, we wanted to provide an up-to-date overview of the best quality summarized evidence on effects of all types of interventions aimed at preventing self-harm and suicide.

### Objective

The objective of this review is to summarize the effects of interventions aimed at preventing self-harm and suicide in children and adolescents.

## Methods

This review was registered with the international prospective register of systematic reviews (PROSPERO; CRD42019117942) on February 8 2019.

### Inclusion and exclusion criteria

We included systematic reviews published in 2012 and later (last date searched August 2018), and fulfilling the DARE-criteria ^23^. This five-year cut-off is pragmatic in considering that older reviews are no longer a reliable basis for updated evidence. A review published earlier than 2012 may not include primary studies published the last >10 years. Furthermore, to include the broadest possible evidence base, we included reviews in all the languages available to us: English, Norwegian, Danish or Swedish. The other inclusion criteria (PICO) are presented in Box 1.

Box 1.

**Table:** 

Population:	Children and adolescents under 18 with or without an identified risk of developing problems involving self-harm and/or suicide, or those who have already developed these problems.
Intervention:	Any intervention aimed at preventing or reducing self-harm and suicide, including psychological therapy, pharmaceutical interventions, psychosocial interventions, physical activity or nutrition.
Control:	Other relevant interventions, treatment as usual (TAU) or wait list.
Outcome:	All outcomes evaluated in children and youth, including (but not restricted to) self-harm, completed suicide, other health outcomes, quality of life, function, use of health care, attitudes and unwanted effects of interventions.

We excluded systematic reviews that did not meet the criteria for the above-mentioned PICO:

- Children and adolescents with other main-diagnosis, e.g. children admitted to hospitals because of somatic illness at the same time as experiencing depressive symptoms.
- Interventions with the main objective to prevent other mental health problems, such as depression.
- Interventions preventing other behaviours with no direct association with mental health, e.g. interventions targeting smoking cessation.
- Pharmaceutical interventions compared to placebo. This review was conducted to inform decision-making in Norway, and for this purpose only direct comparisons between pharmaceutical treatments were judged to be relevant.

### Literature search

The literature search for this review was completed in August 2018 and is largely based on IN SUM: a database of systematic reviews on effects of child mental health and welfare interventions ^24^. We reviewed all references indexed in IN SUM. IN SUM indexes reviews related to children’s and young people’s mental health from the following databases: Cochrane Database of Systematic Reviews, Campbell Library, PsycINFO, MEDLINE, Embase, Web of Science, Database of Abstracts of Reviews of Effects (DARE) and Evidence Based Mental Health (see extended data ^25^ for a description of the IN SUM search strategy, including search words). Examples of search words were suicid*, selfharm*, selfharm*, intervention*, strategy, therap*, child*, adoles*.

The present overview of systematic reviews was developed following the principles of the Cochrane handbook ^26^. Two researchers independently reviewed all publications indexed in IN SUM (two of the authors: AD or ISM, and/or a research colleague KTH). Supplementing the references found in IN SUM, we also hand-searched for relevant systematic reviews, in the following databases and organizations:

• The Norwegian Institute of Public Health

• The Swedish agency for health technology assessment and assessment of social services (SBU)

• The Norwegian Directorate of Health

• The Danish Health Authority

• The National Institute for Health and Care Excellence (NICE)

All publications judged to meet the inclusion criteria were retrieved in full text. Two researchers (ISM, AA) independently screened and assessed all full text reviews for potential inclusion. In cases of disagreement, we consulted a third person.

### Assessment of overlap between reviews and methodological quality

We sorted all included reviews by population and intervention comparisons (the PICOs). In cases were more than one review addressed the same comparison for the same population, we included the review with the newest search date (and completeness of this search by considering the included primary studies) and the best quality. In considering overlap, the first author (ISM) extracted this information from the reviews, and the second author (AA) double-checked the information. Further, we assessed the methodological quality of the included reviews based on a checklist for systematic reviews (AMSTAR: A MeaSurement Tool to Assess systematic Reviews) ^27^. Two people (ISM, IB) considered each publication independently and decided on the quality through discussions until consensus.

The final decision on which reviews to include was done through agreement between two of the authors (ISM and AA). Table 1 contains documentation on characteristics of the included reviews, including methodological quality.

**Table 1.** Characteristics and methodological quality of the included systematic reviews.

Reference	Intervention searched for in the review	Comparisons included in the present review of systematic reviews *	Quality (AMSTAR X of 11)	Date of search	The authors’ defined study population
Hawton 2015	All types of interventions	**Interventions for existing self-harm: therapeutic assessment** **versus treatment as usual (TAU)** Population: Adolescents, 12–18-year olds, referred for a psychosocial assessment following an episode of self-injury or self-poisoning, irrespective of intent Intervention: Standard psychosocial history and suicide assessment, a review of this information, identification of target problems, considering ways to change them and motivations to do so, and alternative problem-solving strategies Control: Treatment as usual comprised of standard psychosocial history and suicide risk assessment Length of intervention: 1 hour and 40 minutes Follow-up period: 12 and 24 months	11	>January 2015	Children and adolescents >19 years old with a history of at least one episode of self-harm (included self-harm with the intention of suicide)
		**Interventions for existing self-harm: mentalization based therapy** **adapted for adolescents (MBT-A) versus TAU** Population: Adolescents, 12 to 17-year olds, diagnosed with comorbid depression presenting to emergency departments or community psychiatric services following an episode of self-injury or self- poisoning, irrespective of whether suicidal intent was present Intervention: Mentalization based therapy adapted for adolescents involving manualized psychodynamic psychotherapy sessions for both the adolescent and his/her family Control: Treatment as usual comprised of one individual therapeutic session alone comprised of a variety of psychotherapeutic approaches, or a psychosocial assessment Length of intervention: 12 months Follow-up period: 12 months			
		**Interventions for existing self-harm: dialectical behaviour therapy** **adapted for adolescents (DBT-A) versus TAU or enhanced TAU** Population: Adolescents, 12 to 19-year olds, with a history of multiple episodes of self-harm Intervention: Dialectical behaviour therapy specially adapted for adolescents composed of weekly individual therapy sessions, weekly group skills training, weekly sessions of multifamily skills training, family therapy sessions and telephone counselling as required Control: Treatment as usual comprising individual and family sessions provided by a multidisciplinary treatment team, medication management, and hospital or respite care as required Length of intervention: 19 weeks Follow-up period: 16 weeks and 6 months			
		**Interventions for existing self-harm: cognitive behaviour therapy** **(CBT) versus non-directive psychotherapy** Population: Adolescents, 12 to 17-year olds, presenting to paediatric facilities following self-injury in which an intent to die was indicated Intervention: Individual skill-based treatment focused on improving problem solving and affect management skills, as well as cognitive and behavioural strategies and homework assignments to further improve their skills Control: Supportive relationship therapy focused on addressing the adolescent’s mood and behaviour Length of intervention: 1) active treatment for the first three months including six individual sessions and one adjunct family session with two additional family sessions and two crisis sessions available at the therapist’s discretion; 2) maintenance treatment for the remaining three months which included three sessions Follow-up period: 3, 6 and 12 months			
		**Interventions for existing self-harm: developmental group therapy** **versus TAU** Population: Adolescents, 12 to 17-year olds, referred to child and adolescent services following an episode of intentional self-injury or self-poisoning, irrespective of intent Intervention: Manualized developmental group psychotherapy involving elements of cognitive behavioural therapy, social skills training, interpersonal psychotherapy, dialectical behavioural therapy, and group psychotherapy with or without addition to treatment as usual Control: Treatment as usual (i.e. individual counselling, family individual-based interventions such as counselling, family sessions, pharmaceutical treatment) Length of intervention: Acute treatment phase weekly sessions over 6 weeks, followed by weekly or biweekly booster sessions as long as required Follow-up period: 6 and 12 months			
		**Interventions for existing self-harm: other psychotherapeutic** **approaches** (no primary studies identified)			
		**Interventions for existing self-harm: nutrition** No primary studies identified			
		**Interventions for existing self-harm: pharmacological treatment** No primary studies identified			
		**Interventions for existing self-harm: compliance enhancement** **versus TAU** Population: Children and adolescents, 10 to 19-year olds, admitted to the emergency department of a general hospital following an episode of self-injury irrespective of intent, and/or increased risk for suicidality Intervention: a single, one-hour session that reviewed expectations for outpatient treatment as well as addressing factors likely to impede attendance and treatment misconceptions and encouraged both the adolescent and parent to make verbal contract and to attend all treatment sessions. Follow-up phone-calls 1, 2, 4 and 8 weeks after disposition. Control: TAU Length of intervention: 8 weeks Follow-up period: 3 months			
		**Interventions for existing self-harm: home-based family** **intervention versus TAU** Population: Adolescents aged 16 or younger referred to child and adolescent mental health services following an episode of self- poisoning irrespective of intent Intervention: manualized home-based family therapy intervention involving one assessment session and 4 home visits in addition to treatment as usual Control: Treatment as usual Length of treatment: Not stated Follow-up period: 6 months			
		**Interventions for existing self-harm: emergency cards plus TAU** **versus TAU** Population: adolescents in the ages of 12 to 16 admitted to hospital after an episode of self-injury or self-poisoning Intervention: emergency green card in addition to usual care. The green card acted as a passport to re-admission into a paediatric ward at the local hospital Control: standard follow-up including treatment from a clinic or child psychiatry department as required Length of intervention: 12 months Follow-up period: 12 months			
NICE 2004 (CG16) and Appendix A1 2016 (updated search of CG16)	All types of interventions	**Interventions for existing self-harm: assessment of children and** **adolescents at the emergency department** No primary studies identified	10	>April 2016	Participants (aged 8 years old or above) admitted to hospital for treatment of index episode of self-harm (self-harm or self- poisoning, irrespective of motivation). Self- endorsed self-harming behaviour are also included.
		**Interventions for existing self-harm: compliance enhancement** **versus TAU** Population: Children and adolescents, 10 to 19-year olds, admitted to the emergency department of a general hospital following an episode of self-injury irrespective of intent, and/or increased risk for suicidality Intervention: a single, one-hour session that reviewed expectations for outpatient treatment as well as addressing factors likely to impede attendance and treatment misconceptions and encouraged both the adolescent and parent to make verbal contract and to attend all treatment sessions. Follow-up phone-calls 1, 2, 4 and 8 weeks after disposition. Control: TAU Length of intervention: 8 weeks Follow-up period: 3 months			
		**Interventions for existing self-harm: other psychotherapeutic** **approaches** No primary studies identified			
		**Interventions for existing self-harm: pharmacological treatment** No primary studies identified			
		**Interventions for existing self-harm: other psychosocial** **interventions** No primary studies identified			
NICE 2011 (CG133) and Appendix A2 2016 (updated search of CG133)	All types of interventions	**Interventions for existing self-harm: assessment of children and** **adolescents at the emergency department** No primary studies identified	11	>April 2016	Participants (aged 8 years old or above) admitted to hospital for treatment of index episode of self-harm (self-harm or self- poisoning, irrespective of motivation). Self- endorsed self-harming behaviour are also included.
		**Interventions for existing self-harm: other psychotherapeutic** **approaches** No primary studies identified			
		**Interventions for existing self-harm: psychoeducation** No primary studies identified			
		**Interventions for existing self-harm: pharmacological treatment** No primary studies identified			
		**Interventions for existing self-harm: combination therapy** No primary studies identified			
		**Interventions for existing self-harm: postcards versus TAU** Population: Adolescents and young adults over the age of 12 previously admitted to a specialist poisons hospital after self- poisoning. Intervention: Postcards mailed out 1, 2, 3, 4, 6, 8, 10 and 12 months after discharge, and at the participant’s birthday Control: Treatment as usual Length of intervention: 12 months Follow-up period: Post-intervention			
		**Interventions for existing self-harm: other psychosocial** **interventions** No primary studies identified			
NICE 2018	Suicide preventing interventions in different arenas	**School-based suicide prevention programs versus TAU, alternative** **interventions, wait list or no intervention** Population: School-aged children and adolescents between the ages of 10 and 23 and personnel working with young people (in schools and other local arenas) Intervention: School based programs (e.g. Signs of Suicide/SoS, Garrett Lee Smith Youth Suicide Prevention Program/GLS), in which the adolescents and personnel in schools and other local arenas learned about suicide Control: Wait list, alternative interventions (information on posters in the classrooms) or no intervention (counties in which GLS was not implemented) Length of intervention: Not stated Follow-up period: 3 to 12 months	11	>19 ^th^ of October 2018	No restrictions
		**Primary prevention: reducing access to means** No primary studies identified			
		**Primary prevention: local suicide plans** No primary studies identified			
		**Secondary prevention: local approaches to suicide clusters** **versus historical control** Population: Children, adolescents and young adults between the ages of 10 and 24 Intervention: Interventions focusing on how the psychiatric services responded after suicide clusters, including debriefing from clinicians giving information, identifying individuals with an increased risk of self- harm, individual screening, and crisis evaluation Control: Historical Length of intervention: Not stated Follow-up period: 4 years			
		**Primary prevention: local media reporting of suicides in** **newspapers, Internet or other digital channels versus historical** **control** Population: Population based sample, a wider age-range than children and adolescents Intervention: One study examining suicides before or after a news story, the other effects of a new guideline for media reporting of suicides Control: Historical Length of intervention: Not stated Follow-up period: Not stated			
		**Interventions to prevent suicide in residential custodial and** **detention settings** No primary studies identified			
		**Secondary prevention: interventions to support children and** **adolescents bereaved or affected by a suspected suicide versus** **TAU or historical control** Population: Children and adolescents in primary and secondary school (under the age of 17) that have lost a friend or parent to suspected suicide Intervention: Bereavement group intervention, weekly meetings led by a psychologist Control: Treatment as usual (no bereavement group) or historical Length of intervention: 10 weeks Follow-up period: Not stated			
		**Primary prevention: screening for suicide risk versus no** **screening** Population: Adolescents between the ages of 13 and 19 Intervention: Screening of symptoms of depression and a history of self-harm, suicidal ideation or suicide attempts Control: No screening Length of intervention: Not stated Follow-up period: Not stated			
O’Connor 2013	Screening for and treatment of suicide risk	**Interventions for existing self-harm: postcards versus TAU** Population: Adolescents and young adults between the ages of 15 to 24 with a history of suicidal threats, ideation, attempts and/or self- injury who did not meet entry criteria for service because they either were not well enough or were receiving treatment elsewhere Intervention: Postcards mailed out monthly over 12 months expressing interest for that person’s well-being, remining him or her about previously identified sources of help and describing one of six rotating self-help strategies (e.g. physical activity, books, Web-sites) Control: Treatment as usual Length of intervention: 12 months Follow-up period: Post-intervention	8	>June 2013	Adolescents and adults in contact with primary or secondary care, mainly with diagnosis such as depression, boarderline personality disorder, PTSD and/or substance abuse
		**Interventions for existing self-harm: pharmacological treatment** No primary studies identified			
Ougrin 2015	All types of interventions	**Interventions for existing self-harm: pharmacological treatment** No primary studies identified	9	>May 2015	Children and adolescents with a history of at least one episode of self-harm (self-harm or self- poisoning, irrespective of intent)
SBU 2014	School- based universal, selective or indicative suicide prevention programmes	**School-based suicide prevention programs versus TAU, alternative** **interventions, waiting list or no intervention** Population: School aged adolescents between the ages of 13 and 19 Intervention: School based prevention programs Control: Treatment as usual (classes as usual), or alternative interventions (alternative classes) or no interventions (schools where the programs were not implemented) Length of intervention: Not stated Follow-up period: 6 to 12 months, and 15 years	7	>October 2014	Children and adolescents with or without identified increased risk for self- harm and/or suicide
Witt 2017	Digital interventions (self-help)	**Interventions for existing self-harm: digital interventions for** **self-management of suicidal ideation and self-harm versus** **psychoeducation or historical control** Population: Adolescents with self-reported suicidal ideation and/or receiving treatment for depression Intervention: Digital self-management programs (iCBT: Internet-based cognitive behaviour therapy, CATCH-IT: program consisting of 14 modules of CBT, Interpersonal therapy (IPT) and community resiliency activities, LEAP: program informed by the Interpersonal Theory of Suicide/LEAP) Control: Psychoeducation or historical Length of intervention: 2 to 12 weeks Follow-up period: Post-intervention	6	>March 2017	No restrictions

*Due to overlap of intervention comparisons for the same population, we included the review with the newest search (and completeness of this search by considering the included primary studies) and the best quality.

### Data extraction and analyses

ISM extracted data from the systematic reviews and AA checked its accuracy. As this was an overview of systematic reviews, we extracted information as it was reported in the systematic reviews, including any supplementary tables or appendixes. We did not retrieve primary studies to provide additional information about interventions or results.

From the systematic reviews, we extracted information about the primary studies’ populations, characteristics of the interventions and comparison groups, duration of the interventions, follow-up periods, outcome measures and pooled effect estimates for each outcome. In cases were the effect estimates were not pooled in a meta-analysis, we reported the results of each individual study for each outcome. 

We did not attempt any reanalysis, but present results as reported in the systematic reviews. For reviews including studies on both children/adolescents and adult populations, we only extracted information from studies on children and adolescents. When reported, the effect estimates were presented with relevant measures of uncertainty.

### Assessing the certainty of evidence and reporting of results

We assessed our confidence in the evidence of effect for each outcome using the GRADE methodology (the Grading of Recommendations Assessment, Development and Evaluation) ^28^. If the systematic review authors already had completed a GRADE assessment, we reviewed this. We describe our confidence in the effect estimates as high, moderate, low or very low for each outcome.

## Results

### Results of the literature search

**Figure 1.**
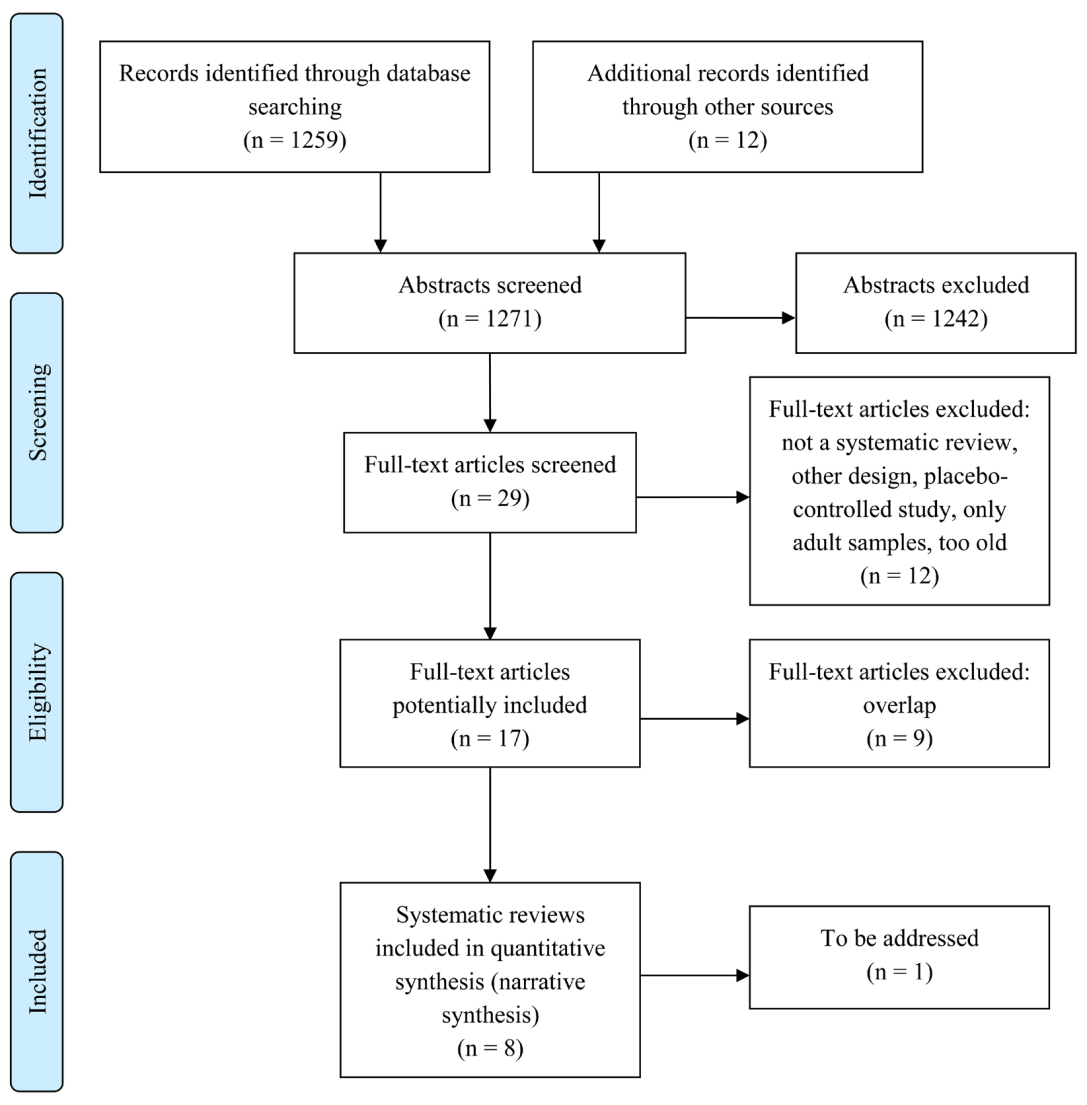
PRISMA flow chart of the study search strategy.

All 1259 references in the INSUM database was reviewed for potential relevance (see Figure 1). Additionally, we identified 12 records through hand-searches. Of the all together 1271 references, we excluded 1242 based on title or summary, mainly because they focused on other diagnosis or problem-areas than self-harm and/or suicide. Overall, 29 full texts were retrieved, 12 were excluded because they did not fulfil the inclusion criteria. Out of 18 potentially included reviews, 9 were excluded because of overlap (see Table 2 for excluded studies).

**Table 2.** Systematic reviews excluded after full text assessment.

Reference	Reason for exclusion
Brauch, AM, Girresch, SK. A review of empirical treatment studies for adolescents non suicidal self-injury. Journal of cognitive psychotherapy. 2012;26:3–18.	Overlap – covered by Hawton 2015
Calear, AL, Christensen, H, Freeman, A, Fenton, K, Grant, JB, van Spijker, B, *et al.* A systematic review of psychosocial suicide prevention interventions for youth. European Child & Adolescent Psychiatry. 2016;25(5):467–82.	Overlap – covered
Corcoran, J, Dattalo, P, Crowley, M, Brown, E, Grindle, L. A systematic review of psychosocial interventions for suicidal adolescents. Children and Youth Services Review. 2011;33(11):2112–18.	Too old
Cusimano, MD, Sameem, M. The effectiveness of middle and high school-based suicide prevention programmes for adolescents: a systematic review. Injury Prevention. 2011;17:43–9.	Too old
Danish Health Authority. Vurdering og visitation af selvmordstruede. Rådgivning til sunhedspersonale [Internet]. Copenhagen: Danish Health Authority; 2007 [retrieved 29.07.2018]. Available from: https://www.sst.dk/da/udgivelser/2007/vurdering-og-visitation-af-selvmordstruede---raadgivning-til- sundhedspersonale	Does not comply with the DARE- criteria and too old
Frey, LM, Hunt, QA. Treatment for suicidal thoughts and behaviour: a review of family-based interventions. Journal of Marital and Family Therapy. 2017;44(1):107–124.	Does not comply with the DARE- criteria
Inagaki, M, Kawashima, Y, Kawanishi, C, Yonemoto, N, Sugimoto, T, Furuno, T, *et al.* Interventions to prevent repeat suicidal behaviour in patiens admitted to an emergency department for a suicide attempt: A meta-analysis. Journal of Affective Disorders. 2015;175:66–78.	Overlap – covered by Hawton 2015
Labelle, R, Pouliot, L, Janelle, A. A systematic review and meta-analysis of cognitive behavioural treatments for suicidal and self-harm behaviours in adolescents. Canadian Psychology/ Psychologie Canadienne. 2015;56(4):368–78.	Overlap – covered by Hawton 2015
Norwegian Directorate of Health. Handlingsplan for forebygging av selvmord og selvskading 2014– 2017 [Internet]. Oslo: The Norwegian Directorate of Health; 2014 [retrieved 29.06.2018]. Available from: https://helsedirektoratet.no/publikasjoner/handlingsplan-for-forebygging-av-selvmord-og- selvskading-20142017	Does not comply with the DARE- criteria
Norwegian Directorate of Health. Ivaretakelse av etterlatte ved selvmord [Internet]. Oslo: The Norwegian Directorate of Health; 2011 [retrieved 29.06.2018]. Available from: https://www. helsedirektoratet.no/tema/selvskading-og-selvmord	Does not comply with the DARE- criteria and too old
Norwegian Directorate of Health. Nasjonale retningslinjer for forebygging av selvmord i psykisk helsevern [Internet]. Oslo: The Norwegian Directorate of Health; 2006 [retrieved 29.06.2018]. Available from: https://www.helsedirektoratet.no/tema/selvskading-og-selvmord	Does not comply with the DARE- criteria and too old
Norwegian Directorate of Health. Veiledende materiell for kommunene om forebygging av selvskade og selvmord [Internet]. Oslo: The Norwegian Directorate of Health; 2017 [retrieved 29.06.2018]. Available from: https://www.helsedirektoratet.no/tema/selvskading-og-selvmord	Does not comply with the DARE- criteria
Ougrin, D, Tranah, T, Leigh, E, Taylor, L, Asarnow, JR. Practitioner review: self-harm in adolescents. Journal of Child Psychology and Psychiatry. 2012;53(4):337–50.	Overlap – covered by Ourgin 2015 (an update of this review and several others)
Ougrin, D, Latif, S. Specific psychological treatment versus treatment as usual in adolescents with self-harm systematic review and meta-analysis. Crisis. 2011;32(2):74–80.	Too old
Perry, Y, Werner-Seidler, A, Calear, AL, Christensen, H. Web-Based and Mobile Suicide Prevention Interventions for Young People: A Systematic Review. Journal of the Canadian Academy of Child & Adolescent Psychiatry/Journal de l.Acade.mie canadienne de psychiatrie de l.enfant et de l.adolescent. 2016;25(2):73–9.	Overlap – covered by Witt 2017
Robinson, J. A systematic review of school-based interventions aimed at preventing, treating, and responding to suicide-related behaviour in young people. Crisis. 2013;34:164–82.	Overlap – covered by SBU 2015
Robinson, J, Hetrick, SE, Martin, C. Preventing suicide in young people: systematic review. Australian and New Zealand Journal of Psychiatry. 2011;45:3–26.	Too old
SBU. Erfarenheter och upplevelser av bemötande och hjälp bland personer med självskadebeteende [Internet]. Stocholm: Swedish agency for health techonogy assessment and assessment of social services (SBU); 2015 [retrieved 29.07.2018]. Available from: http://www.sbu. se/contentassets/4b3a210e262742c9aede925a23889cb5/bemotande_hjalp_sjalvskadebeteende_ 1_201504.pdf	Does not comply with the DARE- criteria
Smedslund, G, Dalsbø, TK, Reinar, LM. Effects of secondary preventive interventions against self- harm [Internet]. Oslo: Norwegian Institute of Public Health; 2016 [retrieved 29.07.2018]. Available from: https://www.fhi.no/publ/2016/effekter-av-sekundarforebyggende-tiltak-mot-villet-egenskade-/	Partly overlap – our review includes Hawton 2015 and SBU 2015, and we excluded Inagaki 2015 and Soomro 2015
Soomro, GM, Kakhi, S. Deliberate self-harm (and attempted suicide). Clinical Evidence. 2015;05(1012):1–30.	Lacks studies on children and adolescents under 18 years old
Wei, Y, Kutcher, S, LeBlanc, JC. Hot idea or hot air: A systematic review of evidence for two marketed youth suicide prevention programs and recommendations for implementation. J Can Acad Child Adolesc Psychiatry. 2015;24(1):5–16.	Overlap – mostly covered by NICE 2018 and SBU 2014

Figure 1 describes the search-process and the number of articles excluded in each step. Eight systematic reviews ^1, 16, 17, 29– 33^, including summary of new evidence of two of them ^34, 35^, were consequently included in the analysis. One review was identified after we had completed the analysis ^36^ and is therefore not included in the present review of systematic reviews.

Although the initial cut-off for age in our population was 18, two of the reviews included studies with young people up to 24 ^29, 30^. These were included because the upper age limit used to define adolescence in research on self-harm and suicides varies between 18 and 25 ^5^.

### Assessment of quality of systematic reviews

The eight included systematic reviews ^1, 16, 17, 29– 35^ were assessed for quality (see Table 1). Overall, the reviews were of high methodological quality, even though some of the reviews lacked *a priori* design, systematic searches for grey literature and assessment of publication bias. We appraised three systematic reviews ^17, 30, 33^ with AMSTAR-scores in the range of 6–8, and the remaining five ^1, 16, 29– 32, 34, 35^ with AMSTAR-scores in the range of 9–11.

### Description of interventions

The reviews included a broad range of interventions. Most of the studies included adolescent populations in the age-range 12 to 18, with some exceptions of samples including younger children or young adults up to the age of 24. Preventive interventions were either focused on primary prevention for mixed-age population based samples (suicide awareness campaigns and other school-based prevention programs, screening for suicide risk) or secondary prevention (local approaches following suicide clusters, suicide prevention in residential custodial and detention settings, interventions to support children and adolescents bereaved or affected by a suspected suicide) ^17, 29, 30^. The reviews also included psychosocial or psychological intervention in cases of existing self-harm (defined as a history of at least one episode of self-harm) (therapeutic assessment, mentalization based therapy, dialectic behaviour therapy, cognitive behaviour therapy, developmental group therapy, compliance enhancement, home-based family intervention, emergency green cards, digital interventions for self-management of suicidal ideation and self-harm, postcards) ^16, 30, 31, 34^.

### Summary of findings

The effects of interventions are presented by type population (young people with or without an identified risk, or with existing self-harm, e.g. a history of at least one episode of self-harm) and by treatment comparison. Our assessment of certainty of evidence corresponds to GRADE-tables in Table 3– Table 16. For comparisons with many outcomes, we report the main outcomes in the present results section. See GRADE-assessments in Table 3– Table 16 for the remaining outcomes.

**Table 3.** GRADE-assessment: School-based suicide prevention programs versus treatment as usual (TAU), alternative interventions, wait list or no intervention.

Population: Children and adolescents between the ages of 10 and 23, as well as personnel working with young people in schools and other arenas Intervention: School-based suicide prevention programs Control: TAU, alternative interventions, wait list or no intervention Based on: NICE 2018 and SBU 2014				
Outcomes	Studies (number of participants)	Effect estimates in control group	Effect estimates in intervention group	Quality of evidence (GRADE)
**Suicidal ideation** – 3- to 12-month follow-up period	5 studies (13936 participants)	221 per 7691	171 per 6241; RR 0.67 (95% KI 0.48 to 0.93)	⊕⊕⊕⊖ ^1^ Moderate
**Suicide attempts** – 3-to 12-month follow-up period	5 studies (14042 participants)	113 per 6951	184 per 7089; RR 0.53 (95% KI 0.36 to 0.80)	⊕⊕⊕⊖ ^1^ Moderate
**Suicide attempts (self-reported)** – ≥2-year follow-up period	1 study (173 000 participants)		1.19 fewer attempts per 1000 adolescents (p=0.53)	⊕⊕⊖⊖ ^2^ Low
**Suicide attempts** – 15-year follow-up period	1 study (500 participants)		RR 0.5 (95% KI 0.3 to 0.9)	⊕⊕⊖⊖ ^1, 3^ Low
**Completed suicide** – 3 year-follow-up period	1 study (2095 participants)		1.33 fewer deaths per 100 000)	⊕⊕⊖⊖ ^2^ Low
**Help-seeking (seeking treatment)** – follow-up period not reported	1 study (376 participants)		RR 0.56 (95% KI 0.30 to 1.05)	⊕⊖⊖⊖ ^1, 4^ Very low
**Help-seeking (using telephone helpline)** – follow-up period not reported	1 study (380 participants)		RR 0.29 (95% KI 0.02 to 4.60)	⊕⊖⊖⊖ ^1, 4^ Very low
**Adverse effects**	4 studies (N=not reported)		No numbers reported, but it is concluded that the findings are contradictory	⊕⊖⊖⊖ ^1, 5, 6^ Very low

1. Downgraded by 1 level due to unclear risk of bias.

2. Downgraded by 2 levels because of study design (observational study).

3. Downgraded by 1 level due to imprecision (only 1 study).

4. Downgraded by 1 level due to imprecision (few incidences).

5. Downgraded by 1 level due to lack of reporting (effect estimates and measure of uncertainty)

6. Downgraded by 1 level due to heterogeneity.

The review authors also searched for research on effects of the following interventions (versus treatment as usual (TAU) or alternative interventions), but studies on children and adolescents under the age of 18 were not identified. These were primary and secondary preventive interventions (reducing access to means, local suicide plans, local media reporting of suicides in newspapers, Internet or other digital channels, suicide prevention in residential custodial and detention settings) ^29^ and interventions targeting existing self-harm (assessment in children and adolescents at the emergency department, psychoeducation, pharmacological treatment or a combination of pharmacological treatment and psychotherapy, nutrition, other psychotherapeutic approaches such as problem-solving therapy, psychodynamic therapy, multi-systemic therapy, supportive therapy, or other psychosocial approaches such as counselling, self-management, respite care, assertive outreach) ^1, 31– 35^.

### Preventive interventions

***School-based suicide prevention programs versus TAU, alternative interventions, wait list or no intervention***. The evidence includes 13 studies with <337 221 children and adolescents aged 10 to 23, as well as personnel in different local arenas working with young people ^17, 29^. In one of the studies, the participants (n=320 500) were habitants in a county in which county-based prevention programs were implemented. These participants included school students and personnel in schools and other local arenas. School-based prevention programs probably reduce suicidal ideation (RR 0.67, 95% KI 0.48 to 0.93, moderate certainty⊕⊕⊕⊖) and suicide attempts (RR 0.53, 95% KI 0.36 to 0.80, moderate certainty⊕⊕⊕⊖) at three to 12 months. Regarding suicide attempts, three studies conclude accordingly at six- and 12-month follow-up period. This effect possibly holds at ≥two- and 15-year follow-up (low certainty⊕⊕⊖⊖). Further, school-based interventions possibly reduce the rate of completed suicides at three-year follow-up (low certainty⊕⊕⊖⊖). Effects on help-seeking and unwanted effects are unclear since the evidence for these outcomes is of very low certainty⊕⊖⊖⊖. See Table 3.

***Primary prevention: local approaches following suicide clusters versus historical control***. The evidence includes three studies with children and adolescents between the ages of 10 and 24 ^29^. Follow-up period was up to four years. The evidence of effects of local approaches following suicide clusters is of very low certainty⊕⊖⊖⊖. See Table 4.

**Table 4.** GRADE-assessment: Primary prevention: local approaches following suicide clusters versus historical control.

Population: Children, adolescents and young adults between the ages of 10 and 24 Intervention: Local approaches to suicide clusters Control: Historical Based on: NICE 2018				
Outcome	Studies (number of participants)	Effect estimates in control group	Effect estimates in intervention group	Quality of evidence (GRADE)
**Suicides** – 4-year follow-up period	2 studies (581 participants)	Study 1: 3 suicides over 5 months pre-intervention; Study 2: 4 suicides over 18 months pre-intervention	No suicides	⊕⊖⊖⊖ ^1, 2^ Very low
**Suicide attempts** – follow-up post- intervention	1 study (N=not reported)	4 suicide attempts pre- interventions	1 suicide attempt	⊕⊖⊖⊖ ^1, 2^ Very low
**Adverse effects**			Not reported	

1. Downgraded by 2 levels due to study design (observational studies).

2. Downgraded by 1 level due to lack of precision (few incidences/short follow-up period).

***Secondary prevention: interventions to support children and adolescents bereaved or affected by a suspected suicide compared to TAU or historical control***. The evidence includes two studies ^29^. However, the evidence of effects of interventions to support children and adolescents bereaved or affected by a suspected suicide is of very low certainty⊕⊖⊖⊖. See Table 5.

**Table 5.** GRADE-assessment: Secondary prevention: interventions to support children and adolescents bereaved or affected by a suspected suicide versus treatment as usual (TAU) or historical control.

Population: Children and adolescents in primary and secondary school (under the age of 17) that have lost a friend or parent to suspected suicide Intervention: Interventions to support children and adolescents bereaved or affected by a suspected suicide Control: TAU or historical Based on: NICE 2018				
Outcomes	Studies (number of participants)	Effect estimates in control group	Effect estimates in intervention group	Quality of evidence (GRADE)
**Suicides** – 3-year follow-up period	1 study (89 participants)	3 per 270 (in the study they counted the whole school- population)	0 per 270; RR 0.14 (95% KI 0.01 to 2.75)	⊕⊖⊖⊖ ^1, 2, 3^ Very low
**Depression** (Children’s Depression Inventory, CDI) – 12-week follow-up period	1 study (75 participants)	Mean 53.9 (SD 7.8)	Mean 44.1 (SD 8.7); Mean difference -9.8 (95% KI -16.01 to -3.59)	⊕⊖⊖⊖ ^1, 2, 3^ Very low
**Anxiety** (The Revised Children’s Manifest Anxiety Scale, RCMAS) – 12-week follow-up period	1 study (75 participants)	Mean 56.5 (SD 10.2)	Mean 39.6 (SD 10.6); Mean difference -16.9 (95% KI -25.9 to -7.9)	⊕⊖⊖⊖ ^1, 2, 3^ Very low
**Post-traumatic stress** (The Childhood Posttraumatic Stress Reaction Index) – 12- week follow-up period	1 study (75 participants)	Mean 17.8 (SD 9.1)	Mean 19.6 (SD 11.4); Mean difference -16.9 (95% KI -5.67 to 9.27)	⊕⊖⊖⊖ ^1, 2, 3^ Very low
**Social adjustment** (The Social Adjustment Inventory for Children and Adolescents, SAICA). – 12-week follow-up period	1 study (75 participants)	Mean 1.8 (SD 0.4)	Mean 1.6 (SD 0.2); Mean difference -0.20 (95% KI -0.47 to 0.07)	⊕⊖⊖⊖ ^1, 2, 3^ Very low
**Parental depression** (scale not reported) – 12-week follow-up period	1 study (75 participants)	Mean 9.7 (SD 4.5)	Mean 11.1 (SD 10.5); Mean difference -1.40 (95% KI -3.53 to 6.33)	⊕⊖⊖⊖ ^1, 2, 3^ Very low
**Adverse effects**			Not reported	

1. Downgraded by 1 level due to risk of bias (no blinding).

2. Downgraded by 1 level due to imprecision (few participants).

3. Downgraded by 1 level due to imprecision (only 1 study).

***Primary prevention: screening for suicide risk versus no screening***. The evidence is based on one review ^30^. The review authors did not identify studies evaluating beneficial effects of screening as a preventive strategy in children or adolescents. They did however identify two studies evaluating adverse effects associated with screening for psychological distress and a history of deliberate self-harm and suicidal ideation in primary care settings. The studies comprised of 2650 adolescents between 13 and 19 years old, and the evidence is of very low certainty⊕⊖⊖⊖. See Table 6.

**Table 6.** GRADE-assessment: Primary prevention: screening for suicide risk versus no screening.

Population: Adolescents between the ages of 13 and 19 Intervention: Screening for suicide risk Control: No screening Based on: O’Connor 2013				
Outcomes	Studies (number of participants)	Effect estimates in control group	Effect estimates in intervention group	Quality of evidence (GRADE)
**Improved health outcomes**			Not reported	
**Adverse effects** – follow-up period not reported	2 studies (2650 participants)		Not reported (described that none of the studies found serious adverse effects of screening)	⊕⊖⊖⊖ ^1, 2, 3, 4^ Very low

1. Downgraded by 1 level due to unclear risk of bias (not reported).

2. Downgraded by 1 level due to imprecision (few incidences).

3. Downgraded by 1 level due to lack of reporting of numbers.

4. Downgraded by 2 levels due to not reported study design.

***Interventions for existing self-harm: therapeutic assessment versus TAU***. The evidence includes one study with 70 adolescents, 12 to 18-year olds referred for a psychosocial assessment following an episode of self-injury or self-poisoning, irrespective of intent ^31^. Length of intervention was one hour and 40 minutes. Follow up was 12 and 24 months. The evidence of effects of therapeutic assessment is of very low certainty⊕⊖⊖⊖. See Table 7.

**Table 7.** GRADE-assessment: Interventions for existing self-harm: therapeutic assessment versus treatment as usual (TAU).

Population: Adolescents, 12 to 18-year olds referred for a psychosocial assessment following an episode of self-injury or self- poisoning irrespective of intent Intervention: Therapeutic assessment Control: TAU Based on: Hawton 2015				
Outcomes	Studies (number of participants)	Effects in control group	Effect estimates in intervention group	Quality of evidence (GRADE)
**Repetition of self-harm** – 12-month follow-up period	1 study (69 participants)	147 per 1000	115 per 1000; OR 0.75 (95 % KI 0.18 to 3.06)	⊕⊖⊖⊖ ^1, 2, 3^ Very low
**Repetition of self-harm** – 24-month follow-up period	1 study (69 participants)	265 per 1000	199 per 1000; OR 0.69 (95 % KI 0.23 to 2.14)	⊕⊖⊖⊖ ^1, 2, 3^ Very low
**Treatment adherence** (attendance to first appointment) – follow-up period not reported	1 study (70 participants)	17 per 35	29 per 35; OR 5.12 (95% KI 1.70 to 15.39) Adolescents in the group receiving therapeutic assessment were statistically more likely to attend the first treatment session	⊕⊖⊖⊖ ^1, 2, 3^ Very low
**Suicide** – follow-up period not reported	1 study (N=not reported)		No numbers were reported, but correspondence with primary study authors confirmed that no participants died by suicide in either group during follow-up	⊕⊖⊖⊖ ^1, 2, 3^ Very low
**Adverse effects**			Not reported	

1. Downgraded by 1 level due to risk of bias (no blinding).

2. Downgraded by 1 level due to imprecision (few participants).

3. Downgraded by 1 level due to imprecision (only 1 study).

***Interventions for existing self-harm: mentalization based therapy (MBT-A) versus TAU***. The evidence includes one study with 80 adolescents, 12 to 17-year olds, diagnosed with depression and presenting to emergency departments or community psychiatric services following an episode of self-injury or self-poisoning, irrespective of whether suicidal intent was present ^31^. Length of treatment was 12 months, and follow-up period was also 12 months. The evidence of effects of therapeutic assessment is of very low certainty⊕⊖⊖⊖. See Table 8.

**Table 8.** GRADE-assessment: Interventions for existing self-harm: mentalization based therapy adapted for adolescents (MBT-A) versus treatment as usual (TAU).

Population: Adolescents, 12 to 17-year olds, diagnosed with comorbid depression presenting to emergency departments or community psychiatric services following an episode of self-injury or self-poisoning, irrespective of whether suicidal intent was present Intervention: Mentalization based therapy for adolescents (MBT-A) Control: TAU Based on: Hawton 2015				
Outcomes	Studies (number of participants)	Effects in control group	Effect estimates in intervention group	Quality of evidence (GRADE)
**Repetition of self-harm** – 12-month follow- up period	1 study (71 participants)	829 of 1000	557 of 1000; OR 0.26 (95 % KI 0.09 to 0.78)	⊕⊖⊖⊖ ^1, 2, 3^ Very low
**Treatment adherence** (number of participants completing all 12 months of treatment) – follow-up period post treatment	1 study (80 participants)	17 of 40	20 of 40; OR 1.35 (95% KI 0.56 to 3.27)	⊕⊖⊖⊖ ^1, 2, 3^ Very low
**Depression** (depression sub-scale of MFQ) – 12-month follow-up period	1 study (80 participants)		Mean difference -2,28 (95% KI -2.81 to -1.75)	⊕⊖⊖⊖ ^1, 2, 3^ Very low
**Suicide** – 12-month follow-up period	1 study (N=not reported)		No numbers were reported, but correspondence with primary study authors confirmed that no participants died by suicide in either the intervention or control arms during follow-up	⊕⊖⊖⊖ ^1, 2, 3^ Very low
**Adverse effects**			Not reported	

1. Downgraded by 1 level due to risk of bias (no blinding).

2. Downgraded by 1 level due to imprecision (few participants/incidences).

3. Downgraded by 1 level due to imprecision (only 1 study).

***Interventions for existing self-harm: dialectical behaviour therapy (DBT-A) versus TAU or enhanced TAU***. The evidence includes two studies with 106 adolescents between the age of 12 and 19 years old with a history of multiple episodes self-harm ^31, 34^. Length of treatment was 19 weeks. Follow-up period was 16 weeks and six months. Based on the available evidence, DBT-A has little or no additional effect on repetition or frequency of self-harm (OR 0.72, 95% KI 0.12 to 4.40, low certainty⊕⊕⊖⊖) compared to (enhanced) treatment as usual. However, DBT-A may have a moderate effect on reduction of suicidal ideation (SMD -0.62, 95% KI -1.07 to -0.16, low certainty⊕⊕⊖⊖). The certainty of evidence for other outcomes is very low⊕⊖⊖⊖. See Table 9.

**Table 9.** GRADE-assessment: Interventions for existing self-harm: dialectical behaviour therapy adapted for adolescents (DBT-A) versus treatment as usual (TAU) or enhanced TAU.

Population: Adolescents, 12 to 19-year olds, with a history of multiple episodes of self-harm Intervention: Dialectical behaviour therapy for adolescents (DBT-A) Control: TAU or enhanced TAU Based on: Hawton 2015				
Outcomes	Studies (number of participants)	Effects in control group	Effect estimates in intervention group	Quality of evidence (GRADE)
**Repetition of self-harm** – between 16 weeks and 6 month follow-up-period	2 studies (105 participants)	151 per 1000	113 per 1000; OR 0.72 (95% KI 0.12 to 4.40)	⊕⊕⊖⊖ ^1, 2^ Low
**Frequency of self-harm** – between 16 weeks and 6 month follow-up-period	2 studies (104 participants)		Mean difference -0.79 (95% KI -2.78 to 1.20)	⊕⊕⊖⊖ ^1, 2^ Low
**Treatment adherence (attendance individual** **therapy sessions) **– between 16 week and 6-month follow-up period	2 studies (106 participants)		Mean attendance to individual therapy sessions was 9.14 in the DBT-A-group (95% KI -4.39 to 22.66)	⊕⊖⊖⊖ ^1, 2, 3^ Very low
**Treatment adherence (attendance family** **therapy sessions)** – between 16 week and 6-month follow-up period	2 studies (106 participants)		Mean attendance to family therapy sessions was 0.93 in the DBT-A- group (95% KI -7.01 to 8.86)	⊕⊖⊖⊖ ^1, 2, 3, 4^ Very low
**Treatment adherence (attendance group** **sessions)** –16 week follow-up-period	1 study (77 participants)		Mean attendance to group sessions was 10.70 in the DBT-A group (95% KI 9.73 to 12.67)	⊕⊖⊖⊖ ^1, 2, 5^ Very low
**Treatment adherence (number of** **medication review meetings)** – 6 month follow-up-period	1 study (29 participants)		Mean attendance to medication review meetings was 0.80 in the DBT-A-group (95 % KI -1.07 to 2.67)	⊕⊖⊖⊖ ^1, 2, 5^ Very low
**Number of telephone contacts received** –16 week follow-up-period	1 study (77 participants)		Mean difference -0.20 (95% KI -2.19 to 1.79)	⊕⊖⊖⊖ ^1, 2, 5^ Very low
**Depression** (depression subscale of MFQ) –16 week follow-up-period	1 study (77 participants)		Mean difference -2.39 (95% KI -5.02 to 0.24)	⊕⊖⊖⊖ ^1, 2, 5^ Very low
**Hopelessness** – between 16 week and 12 month follow-up- period	2 studies (101 participants)		Standardized mean difference -0.13 (95 % KI -0.93 to 0.67)	⊕⊖⊖⊖ ^1, 2, 3^ Very low
**Suicidal ideation** – between 16 week and 12 month follow-up-period	2 studies (100 participants)		Standardized mean difference -0.62 (95% KI -1.07 to -0.16)	⊕⊕⊖⊖ ^1, 2^ Low
**Suicide** – between 16 week and 24-month follow-up period	2 studies (N=not reported)		No numbers were reported, but correspondence with primary study authors confirmed that no participants died by suicide in either group during follow-up	⊕⊖⊖⊖ ^1, 2, 6^ Very low
**Adverse effects**			Not reported	

1. Downgraded by 1 level due to risk of bias.

2. Downgraded by 1 level due to imprecision (few participants).

3. Downgraded by 1 level due to heterogeneity.

4. Downgraded by 1 level due to imprecision (very wide confidence interval).

5. Downgraded by 1 level due to imprecision (only 1 study).

6. Downgraded by 1 level due to imprecision (few incidences).

***Interventions for existing self-harm: cognitive behaviour therapy (CBT) versus non-directive psychotherapy***. The evidence contains one study with 39 adolescents between the age of 12 and 17 presenting to a paediatric general or psychiatric facility following self-injury in which an intent to die was indicated ^31^. Length of treatment was six months. Follow-up period was three, six and 12 months. The certainty of evidence for effects of CBT compared to non-directive psychotherapy is very low⊕⊖⊖⊖. See Table 10.

**Table 10.** GRADE-assessment: Interventions for existing self-harm: individual based cognitive behaviour therapy (CBT) versus non-directive psychotherapy.

Population: Adolescents, 12 to 17-year olds, presenting to paediatric facilities following self-injury in which an intent to die was indicated Intervention: Individual based cognitive behaviour therapy (CBT) Control: Non-directive psychotherapy Based on: Hawton 2015				
Outcomes	Studies (number of participants)	Effect estimates in control group	Effect estimates in intervention group	Quality of evidence (GRADE)
**Repetition of self-harm** – 6-month follow-up period	1 study (39 participants)	111 per 1000	190 per 1000; OR 1.88 (95% KI 0.30 to 11.73)	⊕⊖⊖⊖ ^1, 2, 3, 4^ Very low
**Compliance (number of participants** **completing treatment)** – follow-up period post-intervention	1 study (39 participants)	13 per 18	13 per 21; OR 0.63 (95% KI 0.16 to 2.43)	⊕⊖⊖⊖ ^1, 2, 3, 4^ Very low
**Compliance (number of sessions** **attended)** – between 3- and 6-month follow-up period	1 study (31 participants)		Mean number of sessions attended was 0.20 in the CBT-group (95% KI -1.17 to 1.57)	⊕⊖⊖⊖ ^1, 2, 3, 4^ Very low
**Depression** (scale not reported) **** – 6- month follow-up period	1 study (31 participants)		Mean difference -5.89 (95% KI -16.57 to 4.79)	⊕⊖⊖⊖ ^1, 2, 3, 4^ Very low
**Depression** (scale not reported) **** – 12- month follow-up period	1 study (30 participants)		Mean difference -3.56 (95% KI -10.71 to 3.59)	⊕⊖⊖⊖ ^1, 2, 3, 4^ Very low
**Suicidal ideation** (scale not reported) – 6-month follow-up period	1 study (30 participants)		Mean difference -5.11 (95% KI -30.48 to 20.26)	⊕⊖⊖⊖ ^1, 2, 3, 4^ Very low
**Suicidal ideation** (scale not reported) – 12-month follow-up period	1 study (30 participants)		Mean difference -8.44 (95% KI -29.54 to 12.66)	⊕⊖⊖⊖ ^1, 2, 3, 4^ Very low
**Problem-solving** (SPSI and MEPS) – 6-month follow-up period	1 study (30 participants)		Mean difference (SPSI) 17.88 (95% KI -7.70 to 43.46); Mean difference (MEPS) -0.56 (95% KI -3.31 to 2.19)	⊕⊖⊖⊖ ^1, 2, 3, 4^ Very low
**Problem-solving** (SPSI and MEPS) – 12-month follow-up period	1 study (30 participants)		Mean difference (SPSI) 34.00 (95% KI 12.21 to 55.79); Mean difference (MEPS) -0.45 (95% KI -3.15 to 2.25)	⊕⊖⊖⊖ ^1, 2, 3, 4^ Very low
**Suicide** – 12-month follow-up period	1 study (N=not reported)		No numbers were reported, but correspondence with primary study authors confirmed that no participants died by suicide in either group during follow-up	⊕⊖⊖⊖ ^1, 2, 3, 4^ Very low
**Adverse effects**			Not reported	

1. Downgraded by 2 levels due to serious risk of bias.

2. Downgraded by 1 level due to conflict of interest.

3. Downgraded by 1 level due to imprecision (only 1 study).

4. Downgraded by 1 level due to imprecision (few participants/incidences).

***Interventions for existing self-harm: developmental group therapy versus TAU***. The evidence contains three studies with 487 adolescents, 12 to 17-year olds, referred to child and adolescent services following an episode of intentional self-injury or self-poisoning, irrespective of intent ^31^. The acute treatment phase was six weekly sessions, followed by weekly or biweekly booster sessions for as long as required. Follow-up period was between six and 12 months. Based on the available evidence, the effects of developmental group therapy compared to TAU are uncertain on the following outcomes: repetition of self-harm (six months: OR 1.72 95% KI 0.56-5.24, 12 months: OR 0.80 95% KI 0.22 to 2.97), depression (six months: MD 0.40 95% KI -2.76 to 3.55, 12 months: MD -0.93 95% KI -4.03 to 2.17), suicidal ideation (six months: MD 1.27 95% KI -7.74 to 10.28, 12 months: MD -1.51 95% KI 9.62 to 6.59) or suicide (no suicides). The evidence for all the outcomes is of low certainty⊕⊕⊖⊖. See Table 11.

**Table 11.** GRADE-assessment: Interventions for existing self-harm: developmental group therapy versus treatment as usual (TAU).

Population: Adolescents, 12 to 17-year olds, referred to child and adolescent services following an episode of intentional self- injury or self-poisoning, irrespective of intent Intervention: Developmental group therapy Control: TAU Based on: Hawton 2015				
Outcomes	Studies (number of participants)	Effect estimates in control group	Effect estimates in intervention group	Quality of evidence (GRADE)
**Repetition of self-harm** – 6-month follow-up period	2 studies (430 participants)	726 per 1000	820 per 1000; OR 1.72 (95% KI 0.56 to 5.24)	⊕⊕⊖⊖ ^1, 2^ Low
**Repetition of self-harm** – 12-month follow-up period	3 studies (490 participants)	588 per 1000	533 per 1000; OR 0.80 (95% KI 0.22 to 2.97)	⊕⊕⊖⊖ ^1, 2^ Low
**Depression** (scale not reported) **–**6-month follow-up period	2 studies (420 participants)		Mean difference 0.40 (95% KI -2.76 to 3.55)	⊕⊕⊖⊖ ^1, 2^ Low
**Depression** (scale not reported) **–**12-month follow-up period	3 studies (473 participants)		Mean difference -0.93 (95% KI -4.03 to 2.17)	⊕⊕⊖⊖ ^1, 2^ Low
**Suicidal ideation** (scale not reported) – 6- month follow-up period	2 studies (421 participants)		Mean difference 1.27 (95 % KI -7.74 to 10.28)	⊕⊕⊖⊖ ^1, 2^ Low
**Suicidal ideation** (scale not reported) – 12- month follow-up period	3 studies (471 participants)		Mean difference -1.51 (95 % KI -9.62 to 6.59)	⊕⊕⊖⊖ ^1, 2^ Low
**Suicide** – 6-, 7- and 12-month follow-up period	3 studies (N=not reported)		No suicides	⊕⊕⊖⊖ ^1, 3^ Low
**Adverse effects**			Not reported	

1. Downgraded by 1 level due to risk of bias (lack of blinding).

2. Downgraded by 1 level due to imprecision (wide confidence interval).

3. Downgraded by 1 level due to imprecision (few incidences).

***Interventions for existing self-harm: compliance enhancement versus TAU***. The evidence contains one study of 76 adolescents, 12 to 19-year olds, admitted to the emergency department of a general hospital following an episode of self-injury, irrespective of intent, and/or with an increased risk for suicidality ^31^. Length of treatment was eight weeks. Follow-up period was three months. The evidence of effects of compliance enhancement is of very low certainty⊕⊖⊖⊖. See Table 12.

**Table 12.** GRADE-assessment: Interventions for existing self-harm: compliance enhancement versus TAU.

Population: Children and adolescents, 10 to 19-year olds, admitted to the emergency department of a general hospital following an episode of self-injury irrespective of intent, and/or increased risk for suicidality Intervention: Compliance enhancement plus standard disposition planning Control: TAU (e.g. standard disposition) Based on: Hawton 2015 and NICE short-term management, summary of new evidence 2016				
Outcomes	Studies (number of participants)	Effect estimates in control group	Effect estimates in intervention group	Quality of evidence (GRADE)
**Repetition of self-harm** – 6-month follow-up period	1 study (63 participants)	147 per 1000	104 per 1000; OR 0.67 (95% KI 0.15 to 3.08)	⊕⊖⊖⊖ ^1, 2, 3^, Very low
**Treatment adherence (number of** **participants attending at least one treatment** **session)** – follow-up period post-intervention	1 study (63 participants)	31 per 34	27 per 29; OR 1.31 (95% KI 0.20 to 8.41)	⊕⊖⊖⊖ ^1, 2, 3^ Very low
**Treatment adherence (number of sessions** **attended)** – follow-up period post-intervention	1 study (63 participants)		Mean difference 1.30 (95% KI -1.28 to 3.88)	⊕⊖⊖⊖ ^1, 2, 3^ Very low
**Treatment adherence (number of** **participants completing the full course of** **treatment)** – follow-up period post-intervention	1 study (63 participants)	16 per 34	17 per 29; OR 1,59 (95% KI 0.59 to 4.33)	⊕⊖⊖⊖ ^1, 2, 3^ Very low
**Treatment adherence (attendance to** **psychotherapy post discharge)** – follow-up period not reported	1 study (181 participants)		No numbers are reported, but the authors describe that more in the compliance enhancement-group attended psychotherapy	⊕⊖⊖⊖ ^1, 2, 4^ Very low
**Treatment adherence (number of** **participants completing the full course of** **combination treatment (pharmacological** **treatment plus psychotherapy) post-** **discharge)** – follow-up period not reported	1 study (181 participants)		No numbers are reported, but the authors describe that more in the compliance enhancement-group completed the full course of combination treatment	⊕⊖⊖⊖ ^1, 2, 4^ Very low
**Suicide** – 6-month follow-up period	1 study (76 participants)		No participants died by suicide	⊕⊖⊖⊖ ^1, 2, 3^ Very low
**Adverse effects**			Not reported	

1. Downgraded by 1 level due to imprecision (only 1 study).

2. Downgraded by 1 level due to imprecision (few participants).

3. Downgraded by 2 levels due to serious risk of bias.

4. Downgraded by 1 level due to unclear risk of bias.

***Interventions for existing self-harm: home-based family intervention versus TAU***. The evidence contains one study in a sample of adolescents aged 16 years or younger referred to child and adolescent mental health services following an episode of self-poisoning irrespective of intent ^31^. The intervention was a manualized home-based family therapy intervention. Follow-up period was six months. The evidence of effects of home-based family intervention is of very low certainty⊕⊖⊖⊖. See Table 13.

**Table 13.** GRADE-assessment: Interventions for existing self-harm: home-based family intervention versus treatment as usual (TAU).

Population: Adolescents aged 16 years or younger referred to child and adolescent mental health services following an episode of self-poisoning irrespective of intent Intervention: Home-based family interventions plus TAU Control: TAU Based on: Hawton 2015				
Outcomes	Studies (number of participants)	Effect estimates in control group	Effect estimates	Quality of evidence (GRADE)
**Repetition of self-harm** – 6-month follow-up period	1 study (149 participants)	147 per 1000	149 per 1000; OR 1.02 (95% KI 0.41 to 2.51)	⊕⊖⊖⊖ ^1, 2, 3^ Very low
**Treatment adherence (number of** **participants completing the full course of** **treatment)** – follow-up period post-intervention	1 study (161 participants)	28 per 77	39 per 84; OR 1.52 (95% KI 0.81 to 2.85)	⊕⊖⊖⊖ ^1, 2, 3^ Very low
**Hopelessness** (scale not reported) – 6-month follow-up period	1 study (148 participants)		Mean difference 0.20 (95% KI -0.91 to 1.31)	⊕⊖⊖⊖ ^1, 2, 3^ Very low
**Suicidal ideation** (scale not reported) – 6-month follow-up period	1 study (149 participants)		Mean difference -5.10 (95% KI -17.37 to 7.17)	⊕⊖⊖⊖ ^1, 2, 3^ Very low
**Problem-solving** (scale not reported) **** – 6- month follow-up period	1 study (149 participants)		Mean difference -0.30 (95% KI -2.68 to 2.08)	⊕⊖⊖⊖ ^1, 2, 3^ Very low
**Suicide** – follow-up period not reported	1 study (N=not reported)		1 completed suicide in the intervention group	⊕⊖⊖⊖ ^1, 2, 3^ Very low
**Adverse effects**				

1. Downgraded by 1 level due to risk of bias (lack of blinding).

2. Downgraded by 1 level due to imprecision (only 1 study).

3. Downgraded by 1 level due to imprecision (few participants/incidences).

***Interventions for existing self-harm: emergency green cards plus TAU versus TAU***. The evidence contains one study with 105 adolescents between the ages of 12 and 16 who were admitted to hospital following an episode of self-injury or self-poisoning ^31^. The intervention was emergency green cards in addition to usual care. The green card acted as a passport to re-admission into a paediatric ward at the local hospital. Length of treatment was 12 months. Follow-up period was 12 months. The evidence of effects of emergency green cards is of very low certainty⊕⊖⊖⊖. See Table 14.

**Table 14.** GRADE-assessment: Interventions for existing self-harm: emergency green cards versus treatment as usual (TAU).

Population: Adolescents aged 16 years or younger who were admitted to hospital following an episode of self-injury or self-poisoning to re-admit themselves to a paediatric ward in the local hospital on demand if they felt suicidal Intervention: Emergency green cards Control: TAU (standard follow-up including treatment from a clinic or child psychiatry department as required) Based on: Hawton 2015				
Outcomes	Studies (number of participants)	Effect estimates in control group	Effect estimates in intervention group	Quality of evidence (GRADE)
**Repetition of self-harm**– 12-month follow -up period	1 study (105 participants)	121 per 1000	64 per 1000; OR 0.50 (95% KI 0.12 to 2.04)	⊕⊖⊖⊖ ^1, 2, 3^ Very low
**Adverse effects**			Not reported	

1. Downgraded by 2 levels due to serious risk of bias.

2. Downgraded by 1 level due to imprecision (only 1 study).

3. Downgraded by 1 level due to imprecision (few participants).

***Interventions for existing self-harm: digital interventions for self-management of suicidal ideation and self-harm versus psychoeducation or historical control***. The evidence contains three studies with 184 adolescents reporting suicidal thoughts and/or receiving treatment for depression ^16^. The interventions spanned from two to 12 weeks and follow-up was post treatment. The evidence of effects of digital interventions for self-management is of very low certainty⊕⊖⊖⊖. See Table 15.

**Table 15.** GRADE-assessment: Interventions for existing self-harm: digital interventions for self-management versus psychoeducation or historical control.

Population: Adolescents with self-reported suicidal ideation or receiving treatment for depression Intervention: Digital interventions for self-management Control: Psychoeducation or historical Based on: Witt 2017				
Outcomes	Studies (number of participants)	Effect estimates in control group	Effect estimates in intervention group	Quality of evidence (GRADE)
**Suicidal ideation**– follow- up period post-intervention	3 studies (184 participants)		Study 1: Standardized mean difference -1.12 (95% KI -1.72 to -0.53); Study 2: OR 0.16 (95% KI 0,03 to 0.75); Study 3: Standardized mean difference -0.50 (95% KI -0.95 to -0.06)	⊕⊖⊖⊖ ^1, 2, 3^ Very low
**Adverse effects**			Not reported	⊕⊖⊖⊖ ^1, 2, 3^ Very low

1. Downgraded by 1 level due to risk of bias.

2. Downgraded by 1 level due to imprecision (few participants).

3. Downgraded by 2 levels due to study design (2 out of 3 studies were observational).

***Interventions for existing self-harm: postcards versus TAU***. The evidence is based on two systematic reviews ^30, 34^. One of the reviews ^34^ included one study with 2300 adolescents and young adults over the age of 12 previously admitted to a specialist poisons hospital after self-poisoning. The other review ^30^ included one study of 165 adolescents and young adults of 15 to 24 years old with a history of suicidal threats, ideation, attempts and/or self-injury who did not meet entry criteria for service because they either were not unwell enough or were receiving treatment elsewhere. Follow-up was post study. The evidence of effects of postcards is of very low certainty⊕⊖⊖⊖. See Table 16.

**Table 16.** GRADE-assessment: Interventions for existing self-harm: postcards versus treatment as usual (TAU).

Population: Adolescents and young adults, 12 to 24-year olds, admitted to hospital after self-poisoning and/or a history of suicide threats, ideation, attempts, and/or deliberate self-harm who did not meet entry criteria for service, because they either were not unwell enough or were receiving treatment elsewhere Intervention: Postcard or postcards plus TAU Control: TAU Based on: NICE long-term management, summary of new evidence from surveillance, 2016 and O’Connor 2013				
Outcome	Studies (number of participants)	Effect estimates in control group	Effect estimates in intervention group	Quality of evidence (GRADE)
**Suicide attempts** **–**12-month follow-up period	2 studies (2465 participants)		Study 1: RR 1.44 (95% KI 0.36 to 5.76); Study 2: reported as statistically significant reduction in suicide attempts per participant and number of attempts	⊕⊖⊖⊖ ^1, 2, 3^ Very low
**Suicidal ideation** **–**12-month follow-up period	1 study (2300 participants)		Study 2: reported as statistically significant reduction in number of persons with suicidal ideation	⊕⊖⊖⊖ ^1, 2, 3, 4^ Very low
**Self-injury (cutting)** **–**12-month follow-up period	1 study (2300 participants)		Study 2: reported as no statistical difference in self-cutting or in number of self-cutting episodes per participant	⊕⊖⊖⊖ ^1, 2, 3, 4^ Very low
**Adverse effects**			Not reported	

1. Downgraded by 1 level due to possible lack of generalizability (Study 2 is an adolescent population in Teheran).

2. Downgraded by 1 level due to unclear risk of bias.

3. Downgraded by 1 level due to lack of reporting effect estimates and measurement of uncertainty.

4. Downgraded by 1 level due to imprecision (only 1 study).

## Discussion

The present paper gives a comprehensive overview of effects of interventions aimed at preventing self-harm and suicide in children and adolescents. We found evidence to suggest that school-based interventions probably prevent suicidal ideation and suicide attempts short term, and possibly suicide attempts long term. The effects of community-based interventions following suicide clusters and local suicide plans are unknown, as are the benefits and harms of screening young people for suicide risk. The effects of most interventions targeting children and adolescents with known self-harm are also unknown. However, low certainty evidence suggests that dialectical behavioural therapy and developmental group therapy are equally as effective on repetition of self-harm as enhanced treatment as usual. In general, the populations are adolescents in the age-range of 12 to 18 years.

### Effects of preventive interventions: summary of findings and implications

Based on the available research, school-based interventions can prevent suicidal ideation and suicide attempts short term (moderate certainty evidence), and possibly suicide attempts long term (low certainty evidence), which should have obvious implications for policy makers.

As regards other preventive strategies, there is a general a lack of research on effects of recommended practices, such as approaches to risk assessment and local suicide plans. Screening for suicide risk as primary prevention may provide the opportunity of early detection, and if precise, offer the opportunity to provide young people at risk with appropriate treatment. However, it is resource demanding, and based on available research, effects of screening children and young people for symptoms of depression and a history of self-harm or suicidal ideation in the general population are unknown, given very low certainty evidence. Local suicide plans are a recommended strategy in some countries ^29, 37^. However, the effects of such plans on preventing self-harm and suicide in children and young people is yet to be evaluated in research. Therefore, when implemented, approaches to risk assessment and screening programs, as well as local suicide plans, should be closely evaluated.

We identified no reviews evaluating the effects of reducing access to means from children and young people specifically, or on how media reporting of suicides affects suicide rates in children and young people. In these instances, studies on interventions targeting the general population could be informative. Such studies suggest that reducing access to means may be an effective strategy ^29^, and that certain forms of media reporting are associated with an increase in suicides ^29^. Guidelines on how to report on suicides is one suggested strategy to address the possible harms of such reporting ^29^.

Suicide clusters, although rare, is a phenomenon of major concern. When faced with potential social contagion following suicide, communities are expected to act to prevent contaigon and clustering. However, based on a few studies, the certainty of evidence for community-based interventions following suicide clusters is very low, as is the evidence on effects of support-interventions in young people bereaved or affected by a suicide in their family or other network. Even so, some recommendations are agreed upon, e.g. provision of information to relevant agencies in the community and providing support for those directly affected or other vulnerable individuals ^38^. However, given that the above-mentioned research is of very low certainty, we suggest that researchers design appropriate observational studies, allowing for enough observations pre- and post-implementation of preventive measures to inform policy.

The reviews we identified also searched for studies targeting young people in residential custodial and detention settings, but no studies were identified. Therefore, effects of interventions in this high-risk population are uncertain.

### Effects of interventions for existing self-harm: summary of findings and implications

Self-harm is a common reason for referral of adolescents in child and adolescent psychiatric services, and often accompanies other psychiatric symptoms presented in such settings. However, based on the available evidence, only two treatment comparisons evaluating psychological therapy provided evidence of their effectiveness (low certainty); dialectical behavioural therapy and developmental group therapy. Both treatments were compared to enhanced TAU (e.g. individual and family sessions, medication management, and hospital or respite care as required), and there was little or no important difference in effect on repetition of self-harm, nor on symptoms of depression. However, of notice, although not statistically significant, there was a substantial higher degree of repetition of self-harm amongst adolescents participating in group developmental therapy compared to those receiving enhanced TAU at six-month follow-up. At 12-month follow-up, there was little or no important effect on self-harm. Clinicians should be aware of this potential short-term adverse effect, but this should be investigated in future studies. However, the findings on beneficial effects are overall promising. It seems that both dialectical behavioural therapy and developmental group therapy, *or* established treatment approaches, are good treatment alternatives.

For remaining interventions targeting self-harm, effects are unknown. It is uncertain which approach to risk assessment of young people after an episode of self-harm is most appropriate given low certainty evidence. Furthermore, the effects of psychoeducation, psychological therapy, psychosocial interventions, digital interventions for self-management and nutrition for treating young people with existing self-harm are unknown, as no studies were identified.

The reviews we included searched for, but did not identify, studies on direct comparisons between different pharmacological treatment alternatives or on the effects of combination therapy (pharmacological treatment plus psychotherapy). The finding that biological factors may be associated with, or even predict, a suicide attempt ^13^ could have implications for research on pharmacological agents.

The evidence of effects of organization of services, such as home-based treatment and use of emergency green cards, is of very low certainty. New research in this area is pertinent, especially for policy makers.

### Limitations

A limitation of overviews of reviews, and consequently of the present paper, is that the analyses are based on secondary reporting of what the review authors interpreted and reported based on the primary studies. It follows that the primary studies may have provided more information than what is reported in the reviews we included. A primary study investigating e.g. treatment attendance would be relevant to a clinician wanting to meet with a client struggling with suicidality regularily in order to build a working alliance. However, if the review authors did not find such an outcome from a primary study relevant, we will have missed this information. Regardless of this limitation, the reader of our overview of reviews could find a particular primary study referenced in the included review, if there is need to check if the primary study investigated other relevant outcomes.

It is also worth noting that the present paper only included reviews of studies where the intervention was to prevent or treat self-harm and suicide in children and adolescents, with exception of a few population-based studies. Self-harm and suicide are associated with other difficulties such as psychosis, depression and anxiety. Therefore, evidence from studies on children and adolescents at risk for or diagnosed with such conditions may provide important direction in decision-making when faces with self-harm and suicide. However, in studies on these conditions, self-harm and suicide are rarely investigated as outcomes ^39– 41^. An exception is research on depression, with low certainty evidence indicating that combination treatment for depression (pharmacological treatment plus psychotherapy) may lead to a reduced risk for suicide ^40^.

## Conclusions

Overall, evidence of moderate to low certainty suggests that school-based suicide prevention programs can prevent suicide and suicide attempts in young people. The effects of community-based interventions following suicide clusters and local suicide plans are uncertain. Furthermore, it is not possible to make any conclusions about the benefits or harms of screening in young people with or without known risk of self-harm and suicide.

When it comes to treatment strategies for young people with existing self-harm, evidence of low certainty suggests that dialectical behavioural therapy and developmental group therapy are equally as effective on repetition of self-harm as enhanced treatment as usual (often individual and/or family psychotherapy). The effects of other interventions specifically targeting self-harm are unknown, because of lack of research or evidence of very low certainty, and should be evaluated. These interventions include mentalization-based psychotherapy, cognitive behavioural therapy and psychodynamic therapy.

Collectively, due to a general lack of research, and in some cases very low certainty evidence, the effects of most interventions are unknown. This has several implications. First and foremost, more research is needed, including studies on children younger than 12 years og age, as well as long-term follow up. Second, when implementing recommended practice with unknown effects, such as approaches to risk assessment, practice should be closely evaluated. With all types of interventions, there is a possibility for adverse effects. Hence, it is crucial to be mindful that our own preventive actions or treatment efforts could contribute to an increased risk for self-harm and suicide, and both adverse as well as beneficial effects should be evaluated. Third, policy makers and health providers should consider evidence from other relevant populations in decision-making, such as studies on adults, as well as studies on conditions associated with self-harm and/or suicidality, e.g. depression and psychosis.

A final implication worth mentioning is related to the scope of the present review of systematic reviews: effects of interventions. In decision-making, knowledge on effects of interventions should be supplemented with other relevant research, such as therapeutic processes influencing the outcome, as well as integrated with clinical expertise and the child’s or adolescent’s and caregiver’s values and preferences ^42, 43^.

## Data availability

### Underlying data

All data underlying the results are available as part of the article and no additional source data are required.

### Extended data

Figshare: Appendix 1 search strategy. https://doi.org/10.6084/m9.figshare.8223842 ^22^

### Reporting guidelines

Figshare: PRISMA checklist for ‘The effects of interventions preventing self-harm and suicide in children and adolescents: an overview of systematic reviews’. https://doi.org/10.6084/m9.figshare.8223863.v1 ^44^

Figshare: PRISMA flow chart for ‘The effects of interventions preventing self-harm and suicide in children and adolescents: an overview of systematic reviews’. https://doi.org/10.6084/m9.figshare.8223875.v1 ^45^

Data are available under the terms of the Creative Commons Zero "No rights reserved" data waiver (CC0 1.0 Public domain dedication).

## References

[1] NICE: Self-harm in over 8s: Long-term management. Clinical guideline CG133 [Internet]. London: National Institute for Excellence and Health (NICE); 2011. Reference Source 31891461

[2] Hawton K Hall S Simkin S: Deliberate self-harm in adolescents: a study of characteristics and trends in Oxford, 1990-2000. *J Child Psychol Psychiatry.* 2003;44(8):1191–98. 10.1111/1469-7610.00200 14626459

[3] Edmondson AJ Brennan CA House AO: Non-suicidal reasons for self-harm: A systematic review of self-reported accounts. *J Affect Disord.* 2016;191:109–17. 10.1016/j.jad.2015.11.043 26655120

[4] Hawton K Saunders KE O’Connor RC: Self-harm and suicide in adolescents. *Lancet.* 2012;379(9834):2373–82. 10.1016/S0140-6736(12)60322-5 22726518

[5] Bertolote JM Fleischmann A: Suicide and psychiatric diagnosis: a worldwide perspective. *World Psychiatry.* 2002;1(3):181–85. 16946849 PMC1489848

[6] Muehlenkamp JJ Claes L Havertape L: International prevalence of adolescent non-suicidal self-injury and deliberate self-harm. *Child Adolesc Psychiatry Ment Health.* 2012;6:10. 10.1186/1753-2000-6-10 22462815 PMC3348041

[7] Mohl B Skandsen A: The prevalence and distribution of self-harm among Danish high school students. *Personality and Mental Health.* 2012;6(2):147–55. 10.1002/pmh.191

[8] Joiner TE Jr Conwell Y Fitzpatrick KK: Four studies on how past and current suicidality relate even when "everything but the kitchen sink" is covaried. *J Abnorm Psychol.* 2005;114(2):291–303. 10.1037/0021-843X.114.2.291 15869359

[9] Spirito A Esposito-Smythers C: Attempted and completed suicide in adolescence. *Annu Rev Clin Psychol.* 2006;2:237–66. 10.1146/annurev.clinpsy.2.022305.095323 17716070

[10] Lilley R Owens D Horrocks J: Hospital care and repetition following self-harm: multicentre comparison of self-poisoning and self-injury. *Br J Psychiatry.* 2008;192(6):440–5. 10.1192/bjp.bp.107.043380 18515895

[11] Nock MK Borges G Bromet EJ: Suicide and suicidal behavior. *Epidemiol Rev.* 2008;30(1):133–54. 10.1093/epirev/mxn002 18653727 PMC2576496

[12] Wasserman D Cheng Q Jiang GX: Global suicide rates among young people aged 15-19. *World Psychiatry.* 2005;4(2):114–20. 16633527 PMC1414751

[13] Pompili M Gibiino S Innamorati M: Prolactin and thyroid hormone levels are associated with suicide attempts in psychiatric patients. *Psychiatry Res.* 2012;200(2-3):389–394. 10.1016/j.psychres.2012.05.010 22748186

[14] Pompili M Shrivastava A Serafini G: Bereavement after the suicide of a significant other. *Indian J Psychiatry.* 2013;55(3):256–263. 10.4103/0019-5545.117145 24082246 PMC3777347

[15] Molina N Viola M Rogers M: Suicidal ideation in bereavement: a systematic review. *Behav Sci (Basel)* 2019;9(5): pii: E53. 10.3390/bs9050053 31091772 PMC6562884

[16] Witt K Spittal MJ Carter G: Effectiveness of online and mobile telephone applications ('apps') for the self-management of suicidal ideation and self-harm: a systematic review and meta-analysis. *BMC Psychiatry.* 2017;17(1):297. 10.1186/s12888-017-1458-0 28810841 PMC5558658

[17] SBU: Skolebaserade program för att förebygga sjëlvskadebeteende inklusive suicidforsök [Internet].Stocholm: Swedish agency for health techonogy assessment and assessment of social services (SBU);2015 Reference Source

[18] Cusimano MD Sameem M: The effectiveness of middle and high school-based suicide prevention programmes for adolescents: a systematic review. *Inj Prev.* 2011;17(1):43–9. 10.1136/ip.2009.025502 21059602

[19] Frey LM Hunt QA: Treatment For Suicidal Thoughts and Behavior: A Review of Family-Based Interventions. *J Marital Fam Ther.* 2017;44(1):107–124. 10.1111/jmft.12234 28394014

[20] Ougrin D Latif S: Specific psychological treatment versus treatment as usual in adolescents with self-harm: systematic review and meta-analysis. *Crisis.* 2011;32(2):74–80. 10.1027/0227-5910/a000060 21616756

[21] Wei Y Kutcher S LeBlanc JC: Hot Idea or Hot Air: A Systematic Review of Evidence for Two Widely Marketed Youth Suicide Prevention Programs and Recommendations for Implementation. *J Can Acad Child Adolesc Psychiatry.* 2015;24(1):5–16. 26336375 PMC4357329

[22] Ioannidis JP Greenland S Hlatky MA: Increasing value and reducing waste in research design, conduct, and analysis. *Lancet.* 2014;383(9912):166–75. 10.1016/S0140-6736(13)62227-8 24411645 PMC4697939

[23] DARE: Database of abstracts of reviews of effects [Internet]. York: University of York. Center for reviews and dissemination. [retrieved 28.08.18]. Reference Source

[24] IN SUM: a database of systematic reviews on effects of child mental health and welfare interventions [Internet]. Oslo: Regional Centre for Child and Adolescent Mental Health, Eastern and Southern Norway (RBUP). [retrieved 28.08.18]. Reference Source

[25] Morken IS: “Appendix 1 Search Strategy”. *figshare.* 2019 10.6084/m9.figshare.8223842.v1

[26] Higgins JPT Green S (editors): Cochrane Handbook for Systematic reviews of Interventions Version 5.1.0 [Internet]. The Cochrane Collaboration;2011 Reference Source

[27] AMSTAR: assessing the methodological quality of systematic reviews [Internet]. [retrieved 28.08.18]. Reference Source

[28] GRADE: grading of recommendations assessment, development and evaluation [Internet]. [retrieved 28.08.18]. Reference Source

[29] NICE: Preventing suicide in community and custodial settings [Internet].London: National Institute for Excellence and Health (NICE);2018 Reference Source

[30] O’Connor E Gaynes BN Burda BU: Screening for and treatment of suicide risk relevant to primary care: a systematic review for the U.S. Preventive Services Task Force. *Ann Intern Med.* 2013;158(10):741–54. 10.7326/0003-4819-158-10-201305210-00642 23609101

[31] Hawton K Witt KG Taylor Salisbury TL: Interventions for self-harm in children and adolescents. *Cochrane Database Syst Rev.* 2015; (12):CD012013. 10.1002/14651858.CD012013 26688129 PMC8786270

[32] NICE: Self-harm in over 8s: short-term management and prevention of recurrence. Clinical guideline CG16 [Internet]. London: National Institute for Excellence and Health (NICE);2004 Reference Source 31891461

[33] Ougrin D Tranah T Stahl D: Therapeutic interventions for suicide attempts and self-harm in adolescents: systematic review and meta-analysis. *J Am Acad Child Adolesc Psychiatry.* 2015;54(2):97–107.e2. 10.1016/j.jaac.2014.10.009 25617250

[34] NICE: Appendix A.2: summary of new evidence from 4-year surveillance of Self-harm in over 8s: long-term management (2011) NICE guideline CG133 [Internet]. London: National Institute for Excellence and Health (NICE);2016 Reference Source 31891461

[35] NICE: Appendix A.1: summary of new evidence from 12-year surveillance of self-harm in over 8s: short term management and prevention of recurrence (2004) NICE guideline CG16 [Internet]. London: National Institute for Excellence and Health (NICE);2016 Reference Source 31891461

[36] McCabe R Garside R Backhouse A: Effectiveness of brief psychological interventions for suicidal presentations: a systematic review. *BMJ Psychiatry.* 2018;18(1):120. 10.1186/s12888-018-1663-5 29724203 PMC5934886

[37] Norwegian Directorate of Health: Veiledende materiell for kommunene om forebygging av selvskade og selvmord [Internet]. Oslo: The Norwegian Directorate of Health;2017 Reference Source

[38] Public Health England: Identifying and responding to suicide clusters and contagion. A practice resource [Internet]. London: Public Health England;2015 Reference Source

[39] Dahlgren A Hammerstrøm K Bjørndal A: Kunnskapsoppsummering: Effekt av tiltak for angstlidelser hos barn og unge [Internet]. Oslo: Regional Centre for Child and Adolescent Mental Health, Eastern and Southern Norway;2018 Reference Source

[40] Dahlgren A Hammerstrøm K Bjørndal A: Kunnskapsoppsummering: Effekt av tiltak ved depresjon hos barn og unge [Internet]. Oslo: Regional Centre for Child and Adolescent Mental Health, Eastern and Southern Norway;2018 Reference Source

[41] Dahlgren A Morken IS Karlsen K: Kunnskapsoppsummering: Effekt av tiltak ved psykoselidelser hos barn og unge [Internet]. Oslo: Regional Centre for Child and Adolescent Mental Health, Eastern and Southern Norway;2018 Reference Source

[42] Dawes M Summerskill W Glasziou P: Sicily statement on evidence-based practice. *BMC Med Educ.* 2005;5(1):1. 10.1186/1472-6920-5-1 15634359 PMC544887

[43] APA Presidential Task Force on Evidence Based Practice. *Am Psychol.* 2006;61(4):271–85. 10.1037/0003-066X.61.4.271 16719673

[44] Morken IS: “PRISMA Checklist”. *figshare.* 2019 10.6084/m9.figshare.8223863.v1

[45] Morken IS: “PRISMA Flow Chart of Search Strategy”. *figshare.* 2019 10.6084/m9.figshare.8223875.v1

