# Supplementary material for: The effects of interventions preventing self-harm and suicide in children and adolescents: an overview of systematic reviews
Source: F1000Res. 2020 Feb 18;8:890. Originally published 2019 Jun 20. [Version 2] doi: 10.12688/f1000research.19506.2 (PMC7043106; doi:10.12688/f1000research.19506.2)
Supplement: Peer review history

# Peer review: Reviewer response for version 2

**DOI:** 10.5256/f1000research.24717.r60225

**Published:** 2020-02-25

**Authors:** Gianluca Serafini

**Affiliations:** 1 Department of Neuroscience, Rehabilitation, Ophthalmology, Genetics and Maternal and Child Health, University of Genoa, Genoa, Italy

Thank you for asking me to review again the present manuscript. In the revised paper, the authors addressed, in my opinion, most of the major comments raised by reviewers improving both the main structure and quality of the present paper. I have no further additional comments.

I confirm that I have read this submission and believe that I have an appropriate level of expertise to confirm that it is of an acceptable scientific standard.

# Peer review: Reviewer response for version 1

**DOI:** 10.5256/f1000research.21386.r58596

**Published:** 2020-01-15

**Authors:** Gianluca Serafini

**Affiliations:** 1 Department of Neuroscience, Rehabilitation, Ophthalmology, Genetics and Maternal and Child Health, University of Genoa, Genoa, Italy

Thank you very much for asking me to review the present manuscript.

 This is, in summary, an interesting paper aimed to evaluate the effects of interventions preventing self-harm and suicide in children and adolescents in an overview of systematic reviews. The authors reported that (moderate certainty evidence) school-based interventions prevent suicidal ideation and attempts short term, and possibly with long term effects on suicide attempts. Overall, the effects of community-based interventions following suicide clusters and local suicide plans resulted uncertain, as are the benefits and harms of screening young people for suicide risk. In addition, the effects of most interventions targeting children and adolescents with known self-harm were uncertain. They added that (low certainty evidence) dialectical behavioural therapy and developmental group therapy are equally as effective on repetition of self-harm as enhanced treatment as usual.

 The authors may find as follows my main comments/suggestions:

 First, as the authors, throughout the Introduction section, correctly stated that self-harm and suicide are associated with relevant psychosocial impairment and result from underlying factors such as other mental health problems, exposure to traumatic events or other difficult circumstances in the young person’s environment, they might even mention that the emotional turmoil in the case of suicide survivors of patients died by suicide may last a long time, and in some cases, may end with their own suicide. Thus, together with self-harm and suicide, it is fundamental to understand the bereavement process after the suicide of a significant other to provide a proper care, reduce stigma, and improve the outcomes. In addition, specific biological factors such as prolactin and thyroid hormone levels may be dysregulated and significantly associated with self-harm and suicide attempts and even involved in a complex compensatory mechanism to correct reduced central serotonin activity. The assumption that prolactin and thyroid hormones may be associated or even predict a suicide attempt is of great importance given the availability of such data in everyday clinical practice. Physicians of any kind as well as mental health professionals should be aware of the importance to insert as much information possible in the assessment of suicide and self-harm risk. Thus, given the above mentioned information, the authors could include throughout the manuscript, some published papers regarding the mentioned topics (PMID: 24082246; 31091772; 28843902; 22748186; 12866334) ^1^ ^2^ ^3^ ^4^ ^5^.

 In addition, why the authors decided to include all publications in English, Norwegian, Danish or Swedish rather than simply including only studies in English language is a matter of debate and needs to be specified.

 Moreover, the authors should immediately present and discuss, in the first lines of the Discussion section, their most relevant study findings. Conversely, they seem to focus with redundancy on the main aims/objectives of the paper which have been already presented elsewhere.

 Although the authors reported that the present analyses are based on secondary reporting and the interpretation of the review authors as well as that the present report included only reviews of studies where the population was children and young people with existing self-harm, the most relevant limitations/shortcomings of the present study need to be more carefully described for the general readership.

 Finally, what is the take-home message of this manuscript? While the authors stated that practice should be evaluated, and researchers should investigate harmful effects as well as beneficial effects of interventions, they failed, in my opinion, to provide some conclusive remarks about their findings. Here, some further details/information are needed.

I confirm that I have read this submission and believe that I have an appropriate level of expertise to confirm that it is of an acceptable scientific standard, however I have significant reservations, as outlined above.

## References

[1] : Bereavement after the suicide of a significant other. *Indian J Psychiatry*.2013;55(3) : 10.4103/0019-5545.117145 256-63 10.4103/0019-5545.117145 24082246PMC3777347

[2] : Suicidal Ideation in Bereavement: A Systematic Review. *Behav Sci (Basel)*.2019;9(5) : 10.3390/bs9050053 10.3390/bs9050053 31091772PMC6562884

[3] : Relationship between chronobiological thyrotropin and prolactin responses to protirelin (TRH) and suicidal behavior in depressed patients. *Psychoneuroendocrinology*.2017;85: 10.1016/j.psyneuen.2017.07.488 100-109 10.1016/j.psyneuen.2017.07.488 28843902

[4] : Prolactin and thyroid hormone levels are associated with suicide attempts in psychiatric patients. *Psychiatry Res*.2012;200(2-3) : 10.1016/j.psychres.2012.05.010 389-94 10.1016/j.psychres.2012.05.010 22748186

[5] : The neurobiology of suicide and suicidality. *Can J Psychiatry*.2003;48(5) : 10.1177/070674370304800504 292-300 10.1177/070674370304800504 12866334

# Response

**Published:** 2020-02-10

**Authors:** Ida Sund Morken

**Affiliations:** Regional Centre for Child and Adolescent Mental Health, Eastern and Southern Norway, Norway

**1)** First, as the authors, throughout the Introduction section, correctly stated that self-harm and suicide are associated with relevant psychosocial impairment and result from underlying factors such as other mental health problems, exposure to traumatic events or other difficult circumstances in the young person’s environment, they might even mention that the emotional turmoil in the case of suicide survivors of patients died by suicide may last a long time, and in some cases, may end with their own suicide. Thus, together with self-harm and suicide, it is fundamental to understand the bereavement process after the suicide of a significant other to provide a proper care, reduce stigma, and improve the outcomes. In addition, specific biological factors such as prolactin and thyroid hormone levels may be dysregulated and significantly associated with self-harm and suicide attempts and even involved in a complex compensatory mechanism to correct reduced central serotonin activity. The assumption that prolactin and thyroid hormones may be associated or even predict a suicide attempt is of great importance given the availability of such data in everyday clinical practice. Physicians of any kind as well as mental health professionals should be aware of the importance to insert as much information possible in the assessment of suicide and self-harm risk. Thus, given the above mentioned information, the authors could include throughout the manuscript, some published papers regarding the mentioned topics (PMID: 24082246; 31091772; 28843902; 22748186; 12866334).

 

- *Thank you for interesting suggestions. We have added information and some of the recommended citations in the introduction regarding the importance of bereavement as a risk factor in prevention of suicide, as well as in the section on “* *Effects of preventive interventions: summary of findings and implications* *”, and about biological factors as possible risk factors in the introduction, as well as in “* *Effects of interventions for existing self-harm: summary of findings and implications”.*

 

**2) **In addition, why the authors decided to include all publications in English, Norwegian, Danish or Swedish rather than simply including only studies in English language is a matter of debate and needs to be specified.

- *We thank reviewer 2 for pointing this out, and have added information about why choosing English, Norwegian, Danish or Swedish rather than simply including only studies in English language. For pragmatic reasons, we have included languages available to us. Furthermore, guidelines developed in Sweden, Denmark and UK carry out extensive evidence reviews. Neglecting to include these would weaken the evidence base.*

**3) **Moreover, the authors should immediately present and discuss, in the first lines of the Discussion section, their most relevant study findings. Conversely, they seem to focus with redundancy on the main aims/objectives of the paper which have been already presented elsewhere.

- *We agree, and are now more focused on our main findings in the beginning of the discussion. *

**4) **Although the authors reported that the present analyses are based on secondary reporting and the interpretation of the review authors as well as that the present report included only reviews of studies where the population was children and young people with existing self-harm, the most relevant limitations/shortcomings of the present study need to be more carefully described for the general readership.

- *We now described more carefully the most relevant limitations/shortcomings of the present study, including examples of what we mean, so that the limitations become more apparent.*

 

**5) **Finally, what is the take-home message of this manuscript? While the authors stated that practice should be evaluated, and researchers should investigate harmful effects as well as beneficial effects of interventions, they failed, in my opinion, to provide some conclusive remarks about their findings. Here, some further details/information are needed.

- *Thank you, and we agree. We now have the take-home message as well as some further details/information in conclusive remarks about the finding in the conclusion.*

# Peer review: Reviewer response for version 1

**DOI:** 10.5256/f1000research.21386.r52990

**Published:** 2019-11-11

**Authors:** Sze Ngar Vanessa Yuan

**Affiliations:** 1 Child and Adolescent Psychiatry, King's College London, London, UK

The article evaluates the effects of self-harm and suicide prevention interventions for children and adolescents. The rationale behind the study is sufficiently supported by different research. The methodology provided is sufficient except it is unclear why only systematic reviews published in 2012 or later are included. It is also unclear what search terms were used for future replications.

 The authors report a range of outcome measures in the results that may not be directly related to self-harm. Such outcomes (e.g. treatment engagement) were not further commented on or summarised. Overall, the systematic review is very comprehensive but the discussion and summary could be more coherent and with a better flow. For instance, the authors can attempt to comment on the implications of TAU control group versus other active intervention control groups.

I confirm that I have read this submission and believe that I have an appropriate level of expertise to confirm that it is of an acceptable scientific standard.

# Response

**Published:** 2020-02-10

**Authors:** Ida Sund Morken

**Affiliations:** Regional Centre for Child and Adolescent Mental Health, Eastern and Southern Norway, Norway

**1)    **The methodology provided is sufficient except it is unclear why only systematic reviews published in 2012 or later are included. 

· *We thank reviewer 1 for pointing out that this was unclear. This cut-off is pragmatic but similar to that practiced by others, for example, the Cochrane Library (https://community.cochrane.org/editorial-and-publishing-policy-resource/cochrane-review-development/cochrane-review-updates), in considering that an older review is obsolete and no longer a reliable basis for evidence and in need for being updated. It takes time before a review is published, consequently a review may be published one to three years later than the search was done for primary studies. This has improved with time and new publication standards for reviews. Thus, a review published earlier than 2012 may not include primary studies published the last >10 years. A sentence to explain this has now been added to the manuscript.*

**2)    **It is also unclear what search terms were used for future replications.

· *The literature search for this review was completed in August 2018 and is largely based on IN SUM: a database of systematic reviews on effects of child mental health and welfare interventions. We reviewed all references indexed in IN SUM. IN SUM indexes reviews related to children’s and young people’s mental health from the following databases: Cochrane Database of Systematic Reviews, Campbell Library, PsycINFO, MEDLINE, Embase, Web of Science, Database of Abstracts of Reviews of Effects (DARE) and Evidence Based Mental Health. A description of IN SUMs searching strategy is included in extended data. We have now made it explicit that the search-words are included in the IN SUM Search Strategy (extended data, reference 22), we provide examples of search words, and we point out that we screened all the references in IN SUM.*

**3) **The authors report a range of outcome measures in the results that may not be directly related to self-harm. Such outcomes (e.g. treatment engagement) were not further commented on or summarised. 

*·      Thank you for pointing this out, as research on other outcomes are often highly relevant. For this review we included all outcomes as reported by the review authors. Effect estimates and judgements of certainty for each such outcome is reported for all pooled estimates. However, in line with the GRADE recommendations, we only make conclusions on outcomes judged to be of low, moderate or high certainty. When evidence is of very low certainty, the effects of these outcomes are considered to be too uncertain as to make any conclusions.*

**4)** Overall, the systematic review is very comprehensive but the discussion and summary could be more coherent and with a better flow. For instance, the authors can attempt to comment on the implications of TAU control group versus other active intervention control groups.

· *Thank you for this feedback. We have now tried to make the discussion and summary more coherent, see introduction, discussion and summary. We have also commented on the implications of TAU control group versus other active intervention control groups, see* *“* *Effects of interventions for existing self-harm: summary of findings and implications”.*
